# Vitamin D in Ankle Fractures

**DOI:** 10.5704/MOJ.2107.027

**Published:** 2021-07

**Authors:** NK Sinha, RK Sinha

**Affiliations:** 1 Department of Orthopaedics, Manipal University College Malaysia, Malacca City, Malaysia; 2 Department of Obstetrics and Gynaecology, Tata Main Hospital, Jamshedpur, India

Dear editor,

We read with interest the article by Balaji *et al* on predictors of functional outcome in unstable ankle fractures treated surgically^1^. It is of critical importance to know multiple factors that can adversely impact operative outcome following ankle trauma and authors are to be commended for underlining various predicting factors. We believe that one more factor that may be considered in this context is the perioperative Vitamin D level in cases of ankle fracture fixation. A study (n=98) by Warner et al shows a significantly low Foot and Ankle Outcome Score (FAOS) regarding symptoms (p=0.017), activities of daily living (p=0.049) and quality of living (p=0.040) in patients with low serum 25 hydroxy Vitamin D^2^. The study suggests that hypovitaminosis D may be an independent risk factor not confounded by other factors known to adversely impact a surgical outcome. In the study by Moore et al, patients with low Vitamin D were 8.1 times more likely to develop nonunion after ankle reconstruction surgery^3^. It is worth emphasising that various studies show a high prevalence of vitamin D deficiency in orthopaedic trauma cases. In the study on ankle trauma by Warner *et al*, incidence of Vitamin D deficiency (< 20ng/ml) was 37% in patients undergoing ankle fixation while 38% of the patients had vitamin level insufficiency (< 30ng/ml)^2^. In another study by Smith *et al* with a cohort group of 75 ankle and foot cases, 35 of the fracture patients (47%) had an insufficient vitamin D level (below the recommended level of 30ng/ml), and 10 of the patients (13%) had a level that was deficient (< 20ng/ml)^4^. The same study also revealed a low Vitamin D level in the ankle fracture group in comparison to the ankle sprain group. In an article by Bogunovic *et al*, low vitamin D level was found in 34% of patients undergoing foot and ankle surgery and 43% in the 723 study participants who were scheduled to undergo orthopaedic surgery^5^. Another study with 1119 patients showed a high Vitamin D deficiency in orthopaedic trauma cases with mean vitamin D value being 20.57ng/ml^6^. Therefore, it is worth noting that various studies indicate a correlation between vitamin D status and adult fracture healing^7,8^.
